# Bibliometric Analysis of Clinical Trials on the Effect of Sugar Alcohol Consumption on Oral Health: Trends, Insights, and Future Directions (1967-2024)

**DOI:** 10.7759/cureus.60248

**Published:** 2024-05-14

**Authors:** Namrata Dagli, Mainul Haque, Santosh Kumar

**Affiliations:** 1 Karnavati Scientific Research Center, Karnavati School of Dentistry, Karnavati University, Gandhinagar, IND; 2 Pharmacology and Therapeutics, National Defence University of Malaysia, Kuala Lumpur, MYS; 3 Periodontology and Implantology, Karnavati School of Dentistry, Karnavati University, Gandhinagar, IND

**Keywords:** mannitol, sorbitol, xylitol, bibliometric analysis, hydrogenated starch hydrolysates (hsh), oral microbiota, periodontal health, dental health and caries, oral health, sugar alcohol

## Abstract

In recent years, the quest for healthier alternatives to sugar has led to the widespread use of sugar alcohol in various food and beverage products. Sugar alcohols, such as xylitol, sorbitol, and erythritol, are popular substitutes due to their sweet taste and lower calorie content than sucrose. Beyond their role in calorie reduction, sugar alcohols have garnered attention for their potential impact on oral health. The bibliometric analysis of clinical trials on sugar alcohol and oral health in PubMed reveals a dynamic and multifaceted research landscape shaped by various factors. Fluctuations in publication rates over time suggest influences such as shifts in research interests, technological advancements, regulatory changes, and evolving consumer behaviors. Key authors like Makinen KK, Makinen PL, and Soderling E emerge as prolific contributors with collaborative solid networks within the research community. The University of Turku in Finland has been identified as the highest contributing university, while Caries Research is the most contributing journal based on the number of clinical trials published. The country-wise analysis highlights Italy and the United States as substantial contributors, with diverse trajectories of research activity observed across nations. The subject-specific words with the highest cooccurrence are xylitol, dental caries, chewing gum, *Streptococcus mutans*, and saliva. Thematic analysis dives deep into how sugar alcohols relate to oral health, using different methods to study their effectiveness, safety, and how they affect the oral microbiome. The analysis of topic trends indicates ongoing exploration of sorbitol and xylitol, with an increasing emphasis on the potential advantages of xylitol. Additionally, there is notable attention on cariostatic agents, strategies for dental caries prevention, and the emergence of novel research domains like probiotics and erythritol, showcasing the dynamic evolution of oral health research focuses and developments. Overall, this analysis provides valuable insights into the distribution and trends of clinical trial publications, contributing to a nuanced understanding of the research landscape in sugar alcohol and oral health.

## Introduction and background

The relationship between diet and oral health has long been a subject of interest in scientific research and public health discourse [1,2]. Among the various components of the diet, sugar alcohols, also known as polyols, have emerged as notable alternatives to traditional sugars due to their reduced calorie content and minimal impact on blood glucose levels [3,4]. With their ability to provide sweetness without the same caloric impact and potential dental harm as regular sugars, sugar alcohols are often seen as a healthier option [5]. Consequently, they have become increasingly popular as sweetening agents in a broad array of food and beverage items [6,7], including sugar-free gums [8,9], candies [10], and oral care products [11].

Sugar alcohols are carbohydrate molecules resembling sugars and alcohols, derived by replacing hydrogen atoms in sugar molecules with hydroxyl (OH) groups. Unlike ethanol, a type of alcohol, sugar alcohols have multiple hydroxyl groups attached to their carbon backbone [12]. They are classified into natural, synthetic, and naturally occurring types. Natural sugar alcohols like xylitol, erythritol, and sorbitol are found in fruits and vegetables or produced through fermentation or hydrogenation. Synthetic sugar alcohols, such as isomalt, maltitol, and lactitol, are derived from sugar sources through chemical processes. Mannitol is a naturally occurring sugar alcohol in algae and certain fruits [13-15]. Sugar alcohols undergo fermentation by gut bacteria, leading to slower increases in blood sugar levels than sugars. While excessive consumption can cause gastrointestinal discomfort, they are generally considered safe [12]. Sugar alcohols and alcohols like ethanol serve different purposes and have different properties and metabolic pathways despite both containing alcohol functional groups [12,16].

Understanding the effects of sugar alcohol consumption on oral health is of paramount importance, considering the prevalence of dental caries and other oral health issues globally [17-20]. At the same time, sugar alcohols are generally considered safer for dental health than conventional sugars. Their potential impacts on oral microbiota, dental plaque formation, and oral health warrant comprehensive investigation. A bibliometric analysis offers a systematic and quantitative approach to exploring the existing literature on this subject, providing insights into research trends, key contributors, collaboration patterns, and emerging areas of interest. By synthesizing and analyzing a wide array of scholarly publications, a bibliometric study can offer valuable perspectives on the current state of knowledge regarding the impact of sugar alcohol consumption on oral health.

In this study, we aim to conduct a comprehensive bibliometric analysis to uncover trends, patterns, and research gaps concerning sugar and alcohol consumption and its impact on oral health. Expressly, we have set out the following objectives: firstly, to identify and scrutinize the volume and temporal distribution of scholarly publications on sugar alcohol consumption and oral health; secondly, to assess the geographical dispersion of research output and pinpoint leading countries and institutions in this domain; thirdly, to delve into the thematic focus of research articles, examining the specific sugar alcohols studied, oral health outcomes assessed, and methodologies employed; fourthly, to identify influential authors, journals, and research networks that are shaping the discourse on sugar alcohol consumption and oral health; finally, to discuss the implications of existing research findings and identify potential avenues for future investigation and intervention. Through this bibliometric analysis, we aspire to contribute to a deeper understanding of the research landscape concerning the effect of sugar and alcohol consumption on oral health, with significant implications for public health policy and dietary recommendations.

## Review

Materials and methods

Search Strategy and Study Selection

On April 28, 2024, a systematic online search was conducted on PubMed to retrieve relevant literature on sugar alcohol consumption and its effects on oral health. The following search string was used: ("sugar alcohol" OR "Sorbitan" OR "lycasin" OR "glycerol" OR "polyol" OR "Xylitol" OR "sorbitol" OR "mannitol" OR "erythritol" OR "maltitol" OR "isomalt" OR "lactitol" OR "hydrogenated starch hydrolysates" OR "polyglycerol" OR "arabitol") AND ("oral health" OR "Oral tissue" OR "Dental health" OR "Saliva" OR "Dental caries" OR "Xerostomia" OR "Periodont*" OR "Oral microbiome") NOT ("Animals" OR "Ethanol").

Specifically, we considered clinical trials published in English that investigate the consumption of sugar alcohol and its effects on oral health outcomes for inclusion in our study. Studies published in languages other than English are excluded from this analysis. Additionally, animal studies are not considered. We excluded studies that solely focus on the consumption of alcohol or other sugars (e.g., sucrose, fructose, and glucose) without specific mention or analysis of sugar alcohols. Review articles lacking original research data are also excluded. Duplicate publications or studies with overlapping data are also excluded to ensure the integrity and accuracy of the analysis.

Filters available in PubMed were applied to ensure the selection of clinical trials. The data from selected articles were exported to a text file format for further analysis using bibliometric software tools, specifically VOSviewer (developed by Nees Jan van Eck and Ludo Waltman, Centre for Science and Technology Studies, Leiden University) and Biblioshiny (Bibliometrix, developed by Massimo Aria and Corrado Cuccurullo). Subsequently, titles and abstracts of all retrieved articles were manually inspected to assess data reliability. The study selection process was illustrated in a flowchart generated according to the Preferred Reporting Items for Systematic Reviews and Meta-Analyses (PRISMA) guidelines [21].

Data Analysis

Biblioshiny (Rstudio 4.3.1 (Posit PBC, Boston, MA)) was used to extract key information from each article, including publication details (e.g., title, authors, and journal), study characteristics, and main findings. In addition, analysis and visualization of leading contributors and topic trends were also performed [22]. VOSviewer (version 1.6.20) was employed to visualize co-authorship networks and keyword cooccurrence analysis [23]. Microsoft Excel (Microsoft® Corp., Redmond, WA) was used to visualize the data from the analysis conducted using Biblioshiny software. Biorender (BioRender, Toronto, Canada) [24], a graphic design tool, was utilized to create visual representations of the key findings derived from the bibliometric analysis. Descriptive statistics were employed to summarize the findings of the bibliometric analysis. Multiple researchers were involved in the study selection process and data extraction to ensure the accuracy and reliability of the data analysis. Any discrepancies were resolved through discussion and consensus among the research team members.

This bibliometric analysis involved the use of publicly available data from published clinical trials, and no human subjects were directly involved. Therefore, ethical approval was not required for this study. The findings of this bibliometric analysis were compiled into a comprehensive report, presenting graphical representations and interpretive insights to facilitate understanding and dissemination of the results, contributing to the advancement of knowledge in this critical area of healthcare.

Results

Search Results

**Figure 1:**
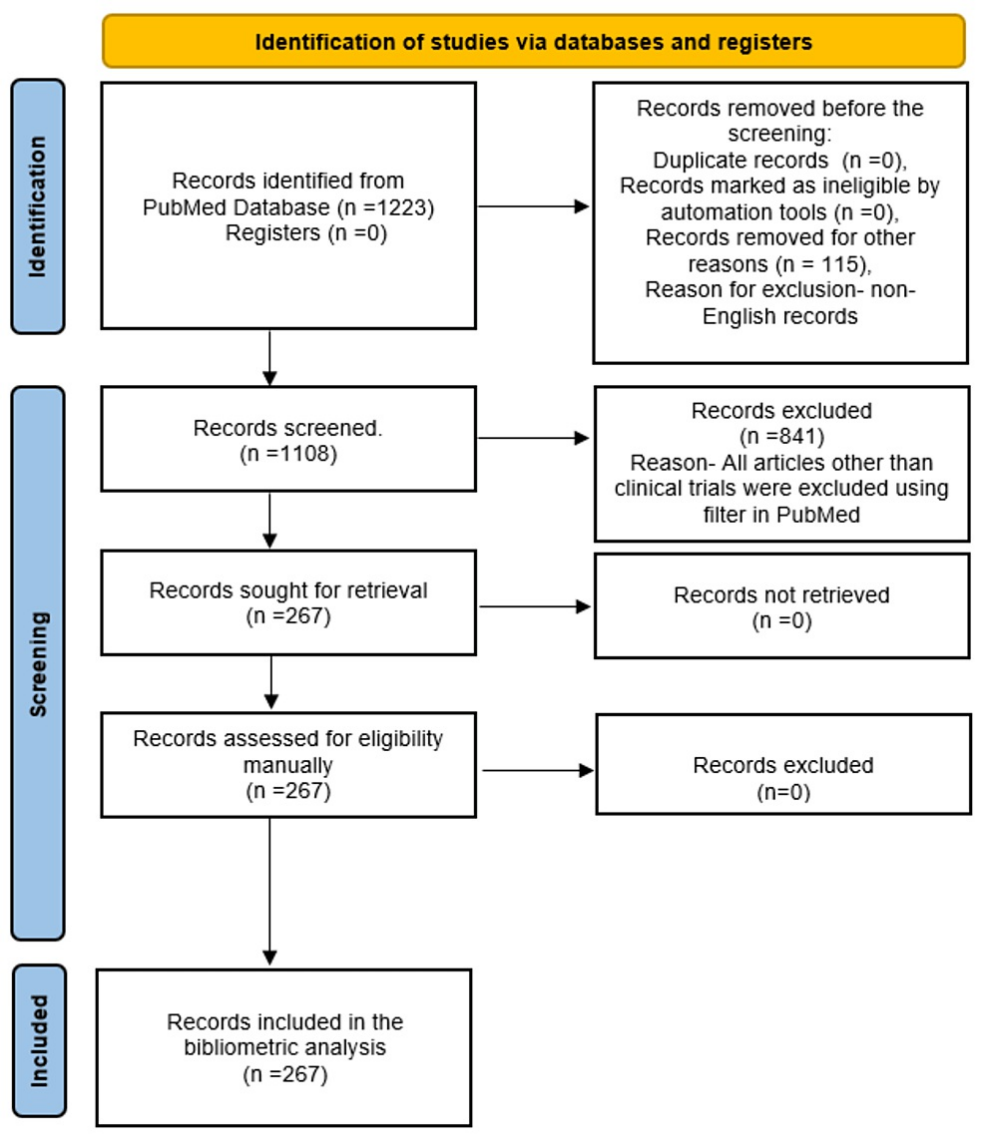
The flowchart depicting the study selection process for the bibliometric analysis of clinical trials on the effect of sugar alcohol consumption on oral health. Image credit: Namrata Dagli

A total of 1,223 publications were identified in the initial search. Among these publications, 1,108 were published in the English language. This subset of English publications includes various studies and documents, including 267 clinical trials, 127 reviews, three case reports, 21 comments, six observational studies, one editorial, and three books or documents. We conducted a thorough manual inspection to ensure the comprehensive inclusion of relevant clinical trials. This meticulous process confirmed the inclusion of 267 clinical trials published in English for further analysis. This selection process and the resulting inclusion of clinical trials in the analysis are illustrated in Figure 1.

Main Information About the Data

According to Biblioshiny, the analysis data included 267 clinical trials on sugar, alcohol, and oral health spanning from 1967 to 2024, published in 95 journals. With an annual growth rate of 1.22%, the literature examined demonstrates a steady expansion over time. Despite the varied publication dates, the average document age is 17.7 years, which suggests that, on average, the documents in the collection are nearly two decades old. Each document, on average, contains one reference, reinforcing the credibility and support for the findings presented. Analysis of the document contents reveals 1,080 keywords. Collaboration among authors is prevalent, with 978 contributing authors and an average of 5.12 co-authors per document. In comparison, most documents result from collaborative efforts; a minority of single-authored works exist, totaling seven documents. International co-authorships, while present, constitute approximately 4.49% of collaborations, suggesting some degree of global engagement in the exploration of sugar alcohol and oral health.

Annual Scientific Production

**Figure 2:**
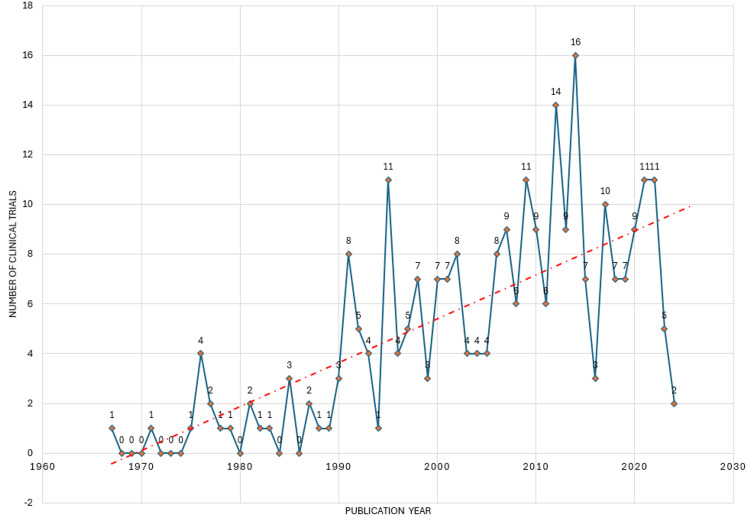
Annual publication of clinical trials on the effect of sugar alcohol consumption on oral health in PubMed. Image credit: Namrata Dagli

The annual publication trend of clinical trials on the effect of sugar alcohol consumption on oral health indicates fluctuations over the years. From the late 1960s to the early 1970s, there were minimal to no publications on this topic. However, interest began to emerge in 1975, with a slight increase in publications. The number of publications remained relatively low throughout the 1980s, with occasional spikes. The 1990s saw a notable uptick, particularly toward the latter half of the decade, with more clinical trials being conducted. This trend continued into the early 2000s, when there were consistent publications each year, often surpassing ten trials annually. The highest peak occurred in 2014, with 16 clinical trials published. Following this peak, the highest decline can be noticed between 2014 and 2016, followed by some years of decreased activity. However, the trendline suggests a sustained interest in researching the relationship between sugar alcohol and oral health, with a moderate to high number of clinical trials conducted annually in recent years (Figure 2).

Most Relevant Authors

**Figure 3:**
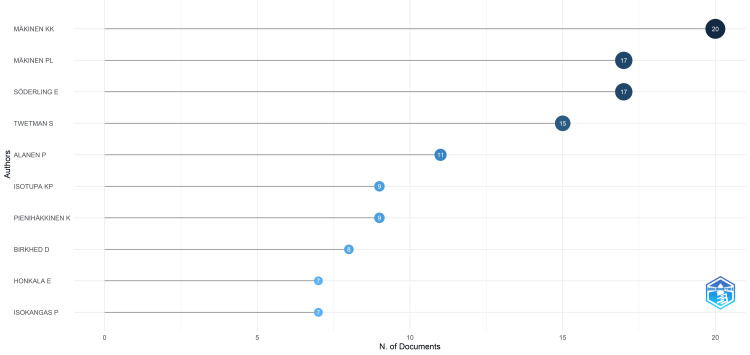
The most relevant authors are based on the number of clinical trials published in PubMed on the effect of sugar and alcohol consumption on oral health. Image credit: Namrata Dagli

Makinen KK stands out with the highest number of articles, 20, followed by Makinen PL and Soderling E, each with 17 articles. Other notable contributors are shown in Figure 3. Based on several publications, all 10 most relevant authors collectively contributed 44.94% of the total clinical trials published in PubMed.

Co-authorship Analysis of Authors

**Figure 4:**
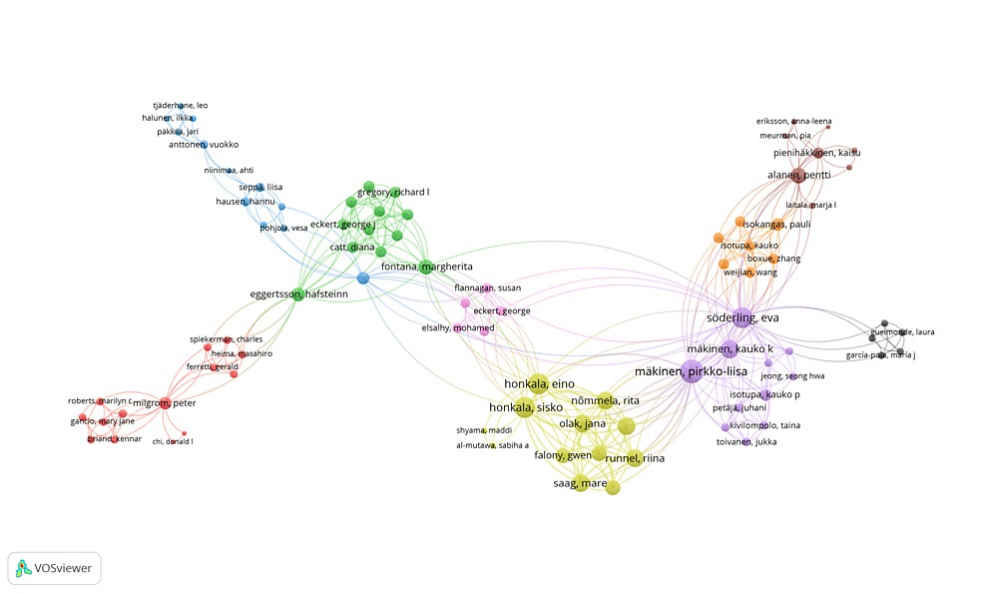
Network visualization of co-authorship analysis of the most prominent set of connected authors. Publication threshold is 1 (weight = total link strength). Cluster 1 is red. Cluster 2 is green. Cluster 3 is blue. Cluster 4 is yellow. Cluster 5 is purple. Cluster 6 is brown. Cluster 7 is orange. Cluster 8 is black. Cluster 9 is pink. Image credit: Namrata Dagli

Out of 1,047 authors identified using VOSviewer, 163 fulfilled the requirement of having two published clinical trials. VOSviewer calculated the total strength of co-authorship links for these 1,047 authors. The largest group of interconnected authors, with one published clinical trial each, comprises 86 authors, depicted in Network Visualization (Figure 4). These authors are distributed across nine clusters, with 381 links and a total link strength (TLS) of 484. Among these authors, Makinen KK stands out with the highest total link strength of 78, with 16 publications, followed by Makinen PL with 59 TLS and 10 publications. The clusters are color-coded as follows: red, green, blue, yellow, purple, brown, orange, black, and pink for Clusters 1 to 9, respectively.

Most Relevant Institutions

In a recent analysis of clinical trial publications, the University of Turku in Finland emerged as the institution with the highest number of publications, totaling 22. The Cincinnati Children's Hospital Medical Center in the United States and the University of Bergen in Norway are closely behind, each with 15 publications. The University of Brescia in Italy and the University of Groningen in the Netherlands tie at 14 publications each, while the University of Ferrara, also in Italy, follows with 11. Other notable institutions include the University of Bari Aldo Moro in Italy, Kagawa University in Japan, Kyushu University in Japan, Umeå University in Sweden, and Universidade Federal do Rio de Janeiro in Brazil, each contributing significantly with 10 or fewer publications. These findings underscore the global reach of research efforts in clinical trials across various countries and continents (Table 1).

**Figure 5:**
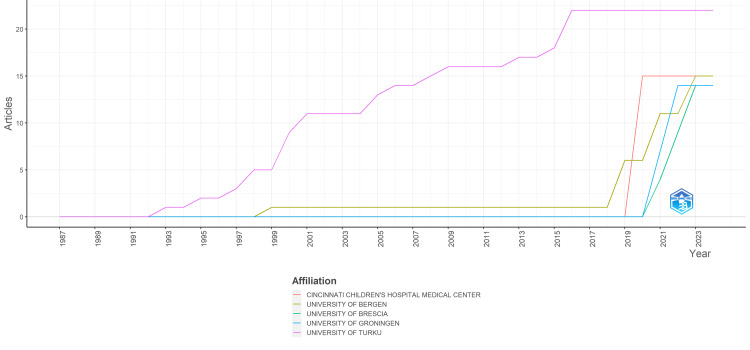
Publications of universities over time of clinical trials on the effect of sugar alcohol consumption on oral health. Image credit: Namrata Dagli

Over the years, the number of clinical trials published by various universities has shown considerable variation. The University of Turku started with no publications in the late 1980s but gradually increased its output, reaching a peak of 22 articles per year from 2016 to 2024. In contrast, the University of Bergen began with minimal activity in the late 1990s, but its research output surged significantly from 2019 onward, with a notable increase to 15 articles per year by 2024. Although starting with no publications, Cincinnati Children's Hospital Medical Center displayed a sudden spike in activity in 2020, maintaining a consistent publication rate of 15 articles per year from 2020 to 2024. The University of Brescia remained dormant until the early 2020s, when it experienced a gradual increase in publications, culminating in 14 articles per year by 2024. The University of Groningen similarly showed no activity until the 2020s but experienced a sharp rise, reaching 14 articles per year by 2022 and maintaining that level through 2024. While some institutions demonstrated consistent growth over time, others experienced sudden bursts of activity, highlighting the dynamic nature of clinical research efforts across different universities (Figure 5).

Co-authorship Analysis of Institutions

**Figure 6:**
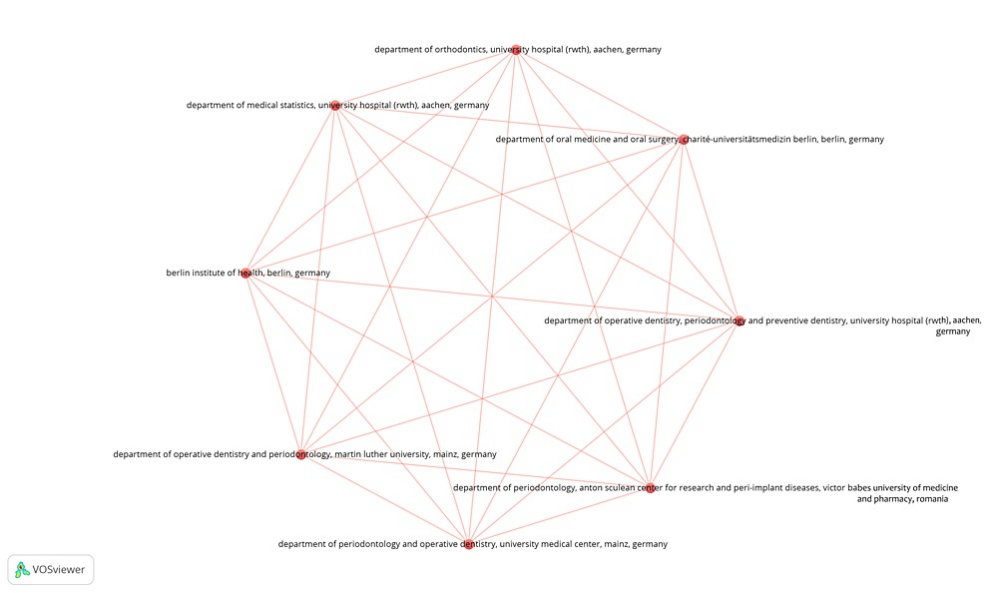
Network visualization of coauthorship analysis of the most extensive set of connected institutions. Weight = total link strength. Image credit: Namrata Dagli

TThree hundred thirty-nine organizations were identified, of which only 20 institutions published two clinical trials in PubMed. For each of the 339 organizations, the total strength of the co-authorship links with the other organizations was calculated. The most extensive connected items included eight institutions (Figure 6). These items are grouped under one cluster with 36 links. Each of these eight organizations published one clinical trial, and each of them had a TLS value of eight.

Most Relevant Sources

Among the top contributors, Caries Research has the highest number of publications, totaling 31. This journal likely serves as a critical platform for research focused on caries prevention and management, which often involves examining the impact of sugar alcohol on dental health. Following closely are Acta Odontologica Scandinavica, Clinical Oral Investigations, and the Journal of Clinical Periodontology, each with 15 publications. While these journals cover a broad spectrum of dental research, their significant contributions likely include investigations into the effects of alcohol consumption on oral health, an area of growing interest due to its potential implications for periodontal diseases and overall oral hygiene. Other notable journals, such as the Journal of Dental Research, BioMed Central (BMC) Oral Health, and the European Journal of Oral Sciences, also demonstrate substantial engagement with these topics, albeit with slightly fewer publications, indicating a diverse landscape of research dissemination within the realm of dental science (Table 2).

**Table 1:** The most relevant journals and the number of clinical trials published by them on the effect of sugar alcohol consumption on oral health in PubMed.

Journals	Number of publications
Caries Research	31
Acta Odontologica Scandinavica	15
Clinical Oral Investigations	15
Journal of Clinical Periodontology	15
Journal of Dental Research	9
BMC Oral Health	7
European Journal of Oral Sciences	7
International Dental Journal	7
Oral Health and Preventive Dentistry	7
Gerodontology	6

**Figure 7:**
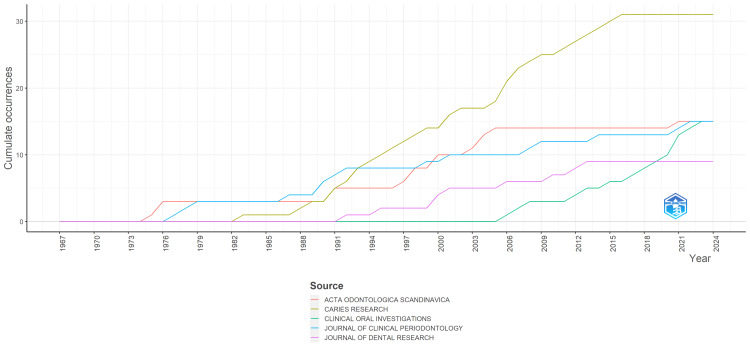
Journal publications over time of clinical trials on the effect of sugar alcohol consumption on oral health. Image credit: Namrata Dagli

The data provided in Figure 7 showcase the evolution of clinical trials published by five prominent journals focusing on sugar, alcohol, and oral health research from 1967 to 2024. Throughout the years, the number of publications varies among the journals. "Caries Research" consistently leads in the number of publications, steadily increasing from zero in the early years to 31 by 2024. "Acta Odontologica Scandinavica" also exhibits a notable upward trend, although not as pronounced as "Caries Research," reaching 15 publications by 2024. "Journal of Clinical Periodontology" and "Journal of Dental Research" show incremental growth over time, with "Journal of Clinical Periodontology" having a slightly higher number of publications compared to "Journal of Dental Research." Interestingly, "Clinical Oral Investigations" remains relatively stable over the years, with minimal fluctuations in the number of publications. Overall, the data reflect the increasing research interest and focus on the relationship between sugar, alcohol, and oral health, as evidenced by the growing number of publications in these leading journals over the decades.

Analysis of Scientific Production of Countries and Collaboration Pattern

**Figure 8:**
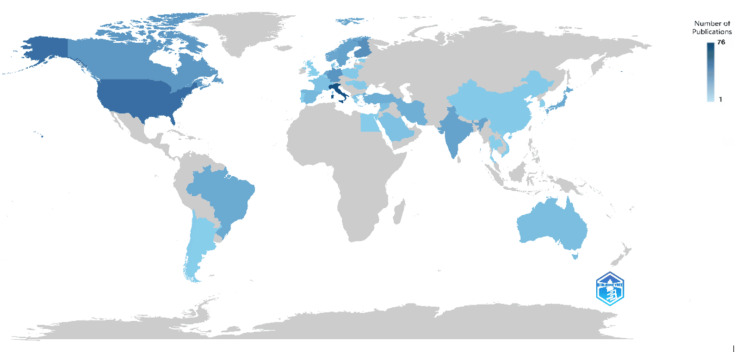
Scientific production of countries of clinical trials on the effect of sugar alcohol consumption on oral health. Image credit: Namrata Dagli

Regarding the number of clinical trials published by different countries, Italy leads the pack with 76 trials, followed closely by the United States with 56 trials. Finland and Germany also demonstrate significant clinical research activity, with 37 and 33 trials, respectively. Canada and India show robust engagement, each contributing 31 and 25 trials, respectively. Sweden and the Netherlands exhibit comparable levels of involvement with 25 and 23 trials, respectively. Norway follows closely with 23 trials, while Brazil and Japan contribute 21. Turkey and Iran demonstrate moderate engagement with 19 and 18 trials, respectively. Spain and Australia show relatively lower activity levels with 13 and 10 trials, respectively. Other countries, such as Syria, France, Saudi Arabia, Denmark, and Switzerland, display varying degrees of involvement with single-digit trial counts. Meanwhile, countries like China, Chile, Egypt, Hungary, Lithuania, Portugal, Argentina, Belgium, Estonia, Israel, South Korea, Thailand, the UK, and Vietnam are represented with only one or a few trials each, indicating comparatively lower levels of participation in published clinical research (Figure 8).

**Figure 9:**
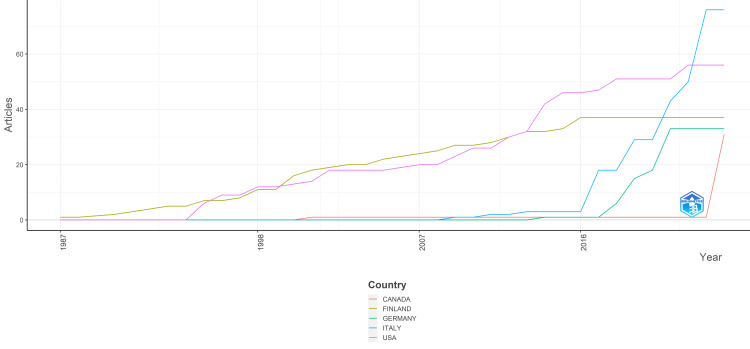
Publications of countries over time of clinical trials on the effect of sugar alcohol consumption on oral health. Image credit: Namrata Dagli

Over the years, there has been a notable variation in the number of clinical trials published by different countries (Figure 9). Finland exhibited a steady increase in clinical trial publications from 1987 onward, with a gradual rise until 2006, followed by a more consistent output, reaching 37 articles annually from 2016 to 2024. In contrast, the United States demonstrated sporadic growth, with no publications until 1995, after which there was a gradual increase, peaking at 56 articles per year from 2022 to 2024. Canada had minimal activity until 2024 when it suddenly surged to 31 articles. Italy showed a similar trend, with negligible publications until 2009, then experiencing a gradual rise, and finally accelerating rapidly from 2017 onward, reaching 76 articles annually from 2023 to 2024. Germany had minimal to no clinical trial publications until 2014, after which there was a gradual increase, particularly notable from 2018 onward, peaking at 33 articles annually from 2021 to 2024. Overall, the United States consistently led in the number of clinical trial publications, followed by Italy, with Finland showcasing a steady but comparatively lower output. Germany and Canada showed minimal activity until recent years when both countries exhibited a notable increase in clinical trial publications.

**Figure 10:**
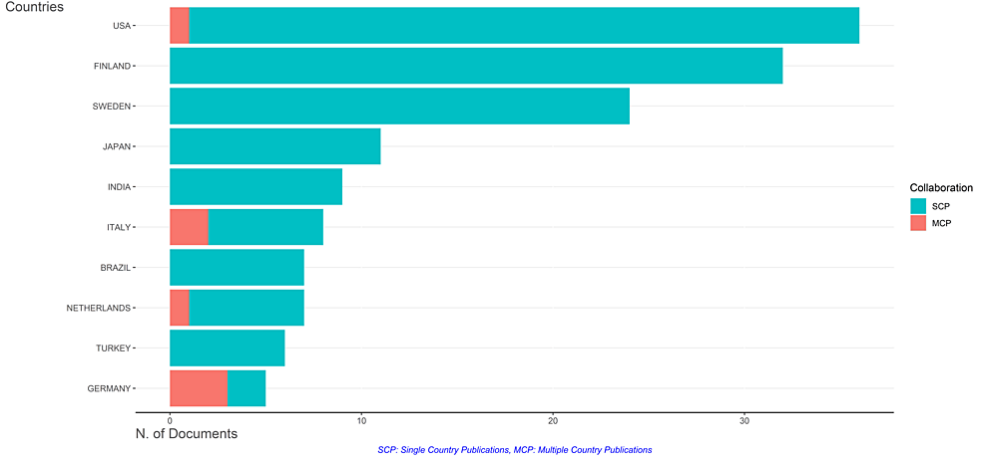
Collaboration pattern of corresponding authors' countries in publishing clinical trials on the effect of sugar alcohol consumption on oral health. Image credit: Namrata Dagli

Over the examined period, the United States consistently led in the number of published clinical trials, with 36 articles. Remarkably, most of these publications were single-country publications, indicating a dominant role in clinical trial research within the country. Finland followed closely behind with 32 articles, all of which were single-country publications, reflecting a focused research landscape within the nation. Sweden maintained a similar trend with 24 articles, all single-country publications. Notably, Japan, India, Brazil, and Turkey each contributed significantly fewer publications, with 11, nine, seven, and six articles, respectively. All were single-country publications, emphasizing a less extensive involvement in multi-country clinical trials. However, Italy and the Netherlands stood out with a notable presence in multi-country publications. Italy contributed eight articles, of which six were single-country publications and two were multi-country publications, while the Netherlands contributed seven articles, with six single-country publications and one multi-country publication. Germany, with fewer total publications with five articles, exhibited a distinctive trend with only two single-country publications but three multi-country publications, suggesting a significant engagement in international collaborative clinical research efforts. While the United States, Finland, and Sweden showcased primarily single-country clinical trials, Italy, the Netherlands, and Germany demonstrated a higher propensity toward multi-country collaborations, reflecting varying degrees of international research engagement among the corresponding authors' countries over time (Figure 10).

Cooccurrence Analysis of Keywords

**Figure 11:**
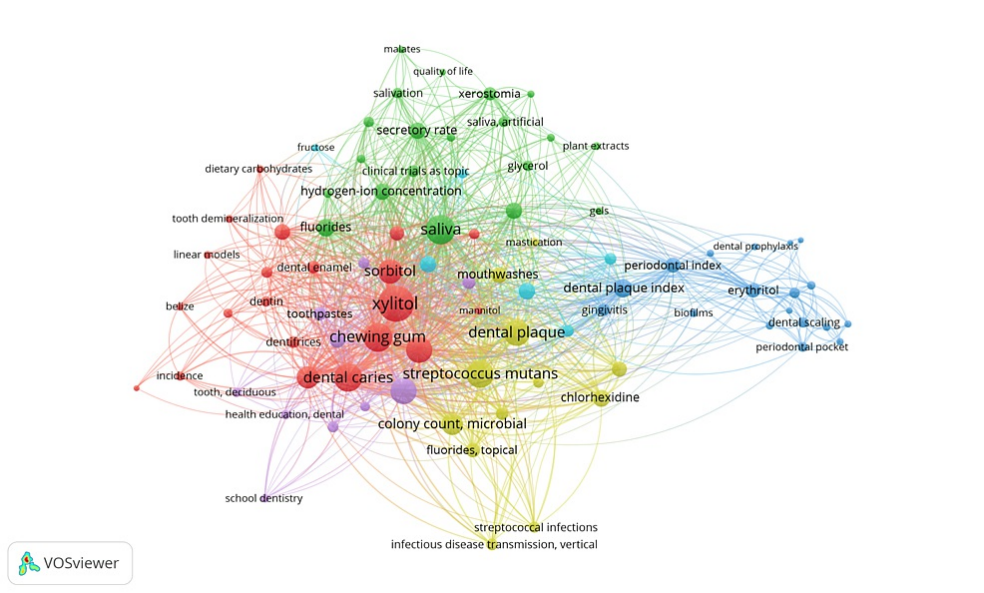
Network visualization of cooccurrence analysis of keywords used in clinical trials on the effect of sugar alcohol consumption on oral health. Weight = total link strength. Each node represents a keyword, and the lines represent the link between them. Cluster 1 is red. Cluster 2 is green. Cluster 3 is blue. Cluster 4 is yellow. Cluster 5 is purple. Cluster 6 is sky blue. Image credit: Namrata Dagli

A total of 624 Medical Subject Headings (MeSH) keywords were identified, of which 124 met the criteria of minimum occurrence of five and 75 met the requirements of minimum occurrence of 10. For each of the 124 words, the total strength of the cooccurrence link with other keywords was calculated by VOSviewer. These keywords were grouped under six clusters, 3,903 links, and 23,199 TLS. To increase the clarity of subject-specific keywords, we excluded nonspecific words related to gender, age, and type of study while generating network visualization of keywords depicted in Figure 11. The subject-specific words with the highest TLS value are xylitol, dental caries, chewing gum, *Streptococcus mutans*, and saliva. Keywords in each cluster are represented in Table 3.

**Table 2:** Keywords in clusters identified in the cooccurrence analysis of keywords. DMF, decay-missing-filled

Clusters	Keywords
Cluster 1 (35 items)	administration oral, adult, aged, aged 80 and over, carboxymethylcellulose, clinical trials as a topic, cross-over studies, drug combinations, female, gels, glycerol, malates, male, middle-aged, mouth mucosa, mouthwashes, oral health, oral hygiene, patient satisfaction, pilot projects, plant extracts, quality of life, random allocation, reference values, root caries, saliva, saliva artificial, salivation, secretory rate, single-blind method, stimulation chemical, surveys and questionnaire, time factors, xerostomia, young adult
Cluster 2 (34 items)	adolescent, analysis of variance, Belize, candy, chewing gum, child, cohort studies, dental caries, dental enamel, dentifrices, dentin, dietary carbohydrates, DMF index, dose-response relationship, double-blind method, Finland, fluorides, health education dental, incidence, linear models, maltose, mannitol, otitis media, risk factors, school dentistry, sorbitol, sugar alcohols, sweetening agents, tooth demineralization, tooth remineralization, tooth deciduous, toothbrushing, toothpaste, xylitol
Cluster 3 (22 items)	biofilms, debridement, dental implants, dental plaque index, dental polishing, dental prophylaxis, dental scaling, erythritol, follow-up studies, gingivitis, humans, periimplantitis, periodontal debridement, periodontal index, periodontal pocket, periodontitis, powders, prospective studies, root planning, surface properties, treatment outcome, ultrasonics
Cluster 4 (18 items)	anti-infective agents local, cariostatic agents, chi-square distribution, child preschool, chlorhexidine, colony count microbial, dental caries susceptibility, fluorides topical, infant, infectious disease transmission, logistic models, longitudinal studies, mothers, mouth, sodium fluoride, statistics nonparametric, streptococcal infections, *Streptococcus mutans*
Cluster 5 (nine items)	the area under curve, buffers, cariogenic agents, dental plaque, hydrogen-ion concentration, mastication, *Streptococcus sobrinus*, sucrose, tablets
Cluster 6 (six items)	bacterial load, fructose, lactic acid, lactobacillus, placebos, probiotics

The keywords in Cluster 1 suggest that research likely includes clinical trials, cross-over studies, and pilot projects focusing on administering oral gels, mouthwashes, and other formulations containing sugar alcohol and its combinations with substances like malates. Malates refer to the salts or esters of malic acid, often utilized in oral health products for their potential therapeutic effects on saliva production or dental caries prevention. It seems to involve both genders, including middle-aged and older adults, with specific attention to those aged 80 and over. Patient satisfaction, quality of life, and xerostomia (dry mouth) might be evaluated using surveys, questionnaires, and assessments of saliva secretion rates and mouth mucosa health. The methodology includes random allocation and single-blind methods, possibly comparing sugar alcohol interventions to reference values or other oral health treatments. Overall, this research aims to investigate the efficacy and safety of sugar-alcohol-based interventions for improving oral hygiene and health outcomes across different age groups and conditions.

Keywords in Cluster 2 include a diverse range of keywords that suggest a multifaceted investigation into the relationship between sugar alcohols and oral health, mainly focusing on children and adolescents. The inclusion of terms like "adolescent," "child," "cohort studies," "dental caries," "dental enamel," "DMF index" (decayed, missing, filled teeth index), and "risk factors" indicates an interest in understanding the prevalence and determinants of dental issues such as tooth decay and demineralization in young populations. Additionally, the presence of terms like "chewing gum," "candy," "sorbitol," "mannitol," and "xylitol" suggests an exploration of sugar alcohols as potential sweetening agents in products like gum and candy, with a focus on their effects on tooth remineralization and demineralization. The mention of "health education dental" implies a consideration of interventions aimed at promoting oral hygiene practices and educating individuals about the impact of dietary carbohydrates, including sugar alcohols, on oral health. Furthermore, including terms like "double-blind method" and "analysis of variance" suggests a methodological rigor in designing and analyzing studies within this research cluster, potentially involving controlled trials and statistical modeling to elucidate dose-response relationships and other factors influencing oral health outcomes. Overall, this cluster indicates a comprehensive investigation into the complex interplay between sugar alcohols, dietary habits, oral hygiene practices, and oral health outcomes in children and adolescents across different populations and settings, encompassing epidemiological and interventional approaches.

Cluster 3, as indicated by the keywords provided, focuses on research on the impact of sugar alcohols, particularly erythritol, on oral health, with a specific emphasis on periodontal and peri-implant conditions. Including biofilms, dental plaque index, periodontal pocket, and periodontitis suggests an investigation into the efficacy of sugar alcohols in combating oral biofilm formation and preventing or treating periodontal diseases. Additionally, terms like dental scaling, root planning, and ultrasonics hint at studies exploring the mechanical aspects of oral hygiene procedures and their interaction with sugar alcohols. The presence of follow-up studies, prospective studies, and treatment outcomes indicates a comprehensive approach to assessing sugar alcohol's long-term effects and effectiveness in dental care, possibly focusing on preventive measures and treatment modalities such as dental prophylaxis and debridement. Overall, the research within this cluster likely seeks to elucidate the role of sugar alcohols, particularly erythritol, in maintaining oral health and preventing periodontal and peri-implant conditions through various interventions and assessments of treatment outcomes.

Cluster 4 keywords suggest research on the relationship between sugar alcohol and oral health, particularly in anti-infective agents, cariostatic agents, and topical fluorides. This research likely explores the effectiveness of various substances, such as chlorhexidine and sodium fluoride, in preventing dental caries, especially in young children (preschool, infant) and their mothers. The presence of streptococcal infections and *Streptococcus mutans* indicates a focus on microbial aspects, possibly involving colony count microbial analysis and longitudinal studies to track changes over time. Statistical methods, such as chi-square distribution and logistic models, may be employed to analyze data, particularly regarding dental caries susceptibility and infectious disease transmission within the mouth. Overall, this research cluster appears to investigate preventive measures and treatments for oral health issues, particularly those related to bacterial infections and dental caries in children and mothers.

The keywords in Cluster 5 suggest a research focus on the interaction between sugar alcohol consumption and oral health, particularly about caries formation and dental plaque. Including terms like "area under the curve" indicates a quantitative approach, possibly involving measurements of sugar alcohol metabolism or its impact on oral pH levels over time. The presence of "buffers" and "hydrogen-ion concentration" suggests an investigation into the buffering capacity of sugar alcohols and their effect on acidity in the oral environment, crucial factors in preventing dental erosion and caries development. "Mastication" hints at a study exploring how sugar alcohol-containing products are chewed and broken down in the mouth, which could influence oral health outcomes. Additionally, *Streptococcus sobrinus* highlights a microbial aspect, suggesting an examination of how sugar alcohols may affect the growth or activity of cariogenic bacteria in dental plaque. These keywords collectively indicate a comprehensive investigation into the potential benefits or risks of sugar alcohol consumption for oral health, encompassing various physiological, microbial, and dietary factors.

The keywords in Cluster 6, "bacterial load," "fructose," "lactic acid," "lactobacillus," "placebos," and "probiotics," suggest a comprehensive investigation into the interplay between sugar alcohols and various factors influencing oral health. The study likely delves into how sugar alcohols affect the microbial environment in the mouth, as indicated by the focus on bacterial load and specific microorganisms like lactobacillus, which can influence dental health. Fructose, a type of sugar commonly found in fruits, may be of interest due to its potential impact on oral bacteria and the production of lactic acid, which can contribute to tooth decay. The inclusion of placebos indicates a possible experimental design involving controlled trials to assess the effects of sugar alcohol on oral health outcomes. Moreover, the mention of probiotics suggests considering interventions aimed at modulating oral microbial populations to promote dental health. Overall, this research likely aims to elucidate the implications of sugar alcohols on oral microbiota and their relevance to maintaining oral health.

Analysis of Topic Trends

**Figure 12:**
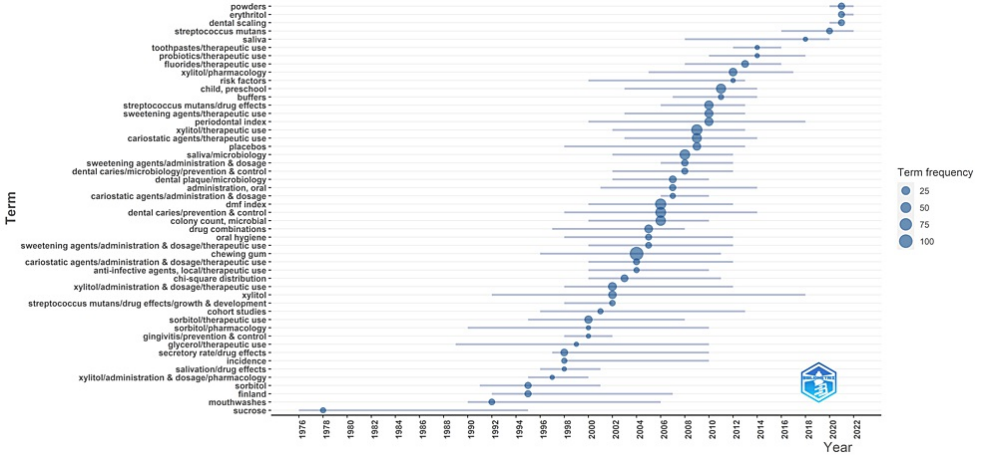
Analysis of topic trends in clinical trials on the effect of sugar alcohol consumption on oral health Field, keywords; frequency threshold, 8 Image credit: Namrata Dagli

Analysis of the frequency of keywords reveals notable patterns over time, as depicted in Figure 12. Research concerning sorbitol and xylitol, two common sugar alcohols, has been consistent throughout the years, with studies focusing on their therapeutic use, administration, and pharmacological effects. Mouthwashes and sorbitol garnered attention from the 1990s to the early 2000s, suggesting a focus on oral hygiene practices and alternative sweeteners. Notably, there has been a significant increase in studies related to xylitol in recent years, indicating a growing interest in its potential benefits for oral health. Additionally, the prominence of terms such as "cariostatic agents," "dental caries prevention," and "*Streptococcus mutans*" suggests a strong emphasis on investigating interventions for preventing dental caries and controlling microbial populations associated with oral health issues. The data also highlight emerging areas of research interest, including probiotics and erythritol, indicating a shift toward exploring novel interventions and alternative sweeteners in oral health research. To increase the clarity of subject-specific keywords, we excluded nonspecific words related to gender, age, and type of study while generating the graph. Overall, the data underscore the dynamic nature of research in this field, reflecting evolving priorities and advancements.

Discussion

The publication trend of clinical trials on sugar alcohol consumption and oral health fluctuated over the years, with minimal activity in the late 1960s to early 1970s, a gradual increase from 1975 onward, notable upticks in the 1990s and early 2000s, peaking in 2014 with 16 trials, followed by a decline between 2014 and 2016, yet showing sustained interest with moderate to high numbers of trials in recent years. The fluctuating pattern in the annual publication of clinical trials on sugar alcohol and oral health suggests a dynamic research landscape influenced by various factors. These include shifts in research interest and funding availability, technological advancements enabling more nuanced studies, changes in regulatory policies or guidelines, evolving consumer behaviors toward sugar-free products, and collaborative efforts between researchers and international initiatives. Peaks in publication rates may coincide with increased awareness of oral health issues, emerging evidence on sugar alcohol effects, or changes in consumer preferences. Understanding these factors can guide future research priorities and interventions promoting oral health and well-being.

Makinen KK emerges as the most prolific author with 20 articles, followed closely by Makinen PL and Soderling E, each contributing 17 articles. These top 10 authors collectively account for a substantial portion, 44.94%, of the total clinical trials published in PubMed, underscoring their significant contribution to the field. Co-authorship analysis further illuminates collaborative networks among authors, with Makinen KK and Makinen PL standing out for their strong connections within the research community.

Institutional analysis reveals the University of Turku in Finland as the leading contributor, with 22 publications, followed by prominent institutions like the Cincinnati Children's Hospital Medical Center in the United States and the University of Bergen in Norway, each with 15 publications. This distribution underscores the global nature of research efforts, with institutions from various countries actively participating in clinical trial research. Moreover, the analysis of publication trends over time highlights dynamic patterns in research output among universities, with some exhibiting consistent growth while others experiencing sudden surges in activity, reflecting the evolving nature of clinical research endeavors.

Caries Research emerges as the foremost contributor, with a consistent increase in publications over the years, indicating its central role in investigations related to caries prevention and the impact of sugar alcohols on dental health. Acta Odontologica Scandinavica, Clinical Oral Investigations, and the Journal of Clinical Periodontology follow closely.

The examination of country-wise scientific production indicates Italy as the frontrunner, with 76 trials, closely trailed by the United States with 56 trials. Noteworthy contributions are also observed from Finland, Germany, Canada, and several other countries, each demonstrating varying levels of engagement in clinical trial publications. Interestingly, while some countries exhibit steady growth in publication output over time, others demonstrate sporadic increases or sudden surges, indicating the diverse trajectories of research activity across different nations.

In a cooccurrence analysis of keywords, the subject-specific terms that stand out prominently due to their high TLS value are xylitol, dental caries, chewing gum, *Streptococcus mutan*s, and saliva. Specifically, this suggests an investigation into the effects of xylitol, a sugar substitute commonly found in chewing gum, on salivation and prevention of dental caries caused by *Streptococcus mutans*. Our literature search also confirms these findings [25-28].

The collective analysis of the keyword clusters reveals a multifaceted exploration into the relationship between sugar alcohols and oral health across different age groups and conditions. These clusters encompass clinical trials, epidemiological studies, and investigations into preventive measures and treatments for oral health issues such as dental caries and periodontal diseases. Various methodologies, including randomized controlled trials and prospective studies, are employed to assess the efficacy and safety of sugar-alcohol-based interventions. Moreover, there's a focus on understanding the microbial dynamics in the oral environment, with attention to specific microorganisms and their interactions with sugar alcohols. The research aims to provide insights into the potential benefits or risks of sugar alcohol consumption for oral health and elucidate mechanisms underlying their effects on oral microbiota and dental outcomes. Analysis of topic trends suggests that there is a consistent exploration of sorbitol and xylitol alongside a growing interest in xylitol's potential benefits, a focus on cariostatic agents and dental caries prevention, and emerging research areas, such as probiotics and erythritol, reflecting the dynamic nature of oral health research priorities and advancements. Our literature search identified several publications on probiotics, erythritol, and microbiome, confirming the findings of the keyword analysis [29-34]. Research suggests that erythritol could be a favorable substitute for sugar in healthy individuals and those with diabetes, as it does not impact glucose or insulin levels [35]. Furthermore, both erythritol and xylitol, given alone or combined, effectively suppress the growth of *Streptococcus mutans* and *Scardovia wiggsiae's* clinical strains while significantly inhibiting biofilm formation by *Streptococcus mutans* [36].

**Figure 13:**
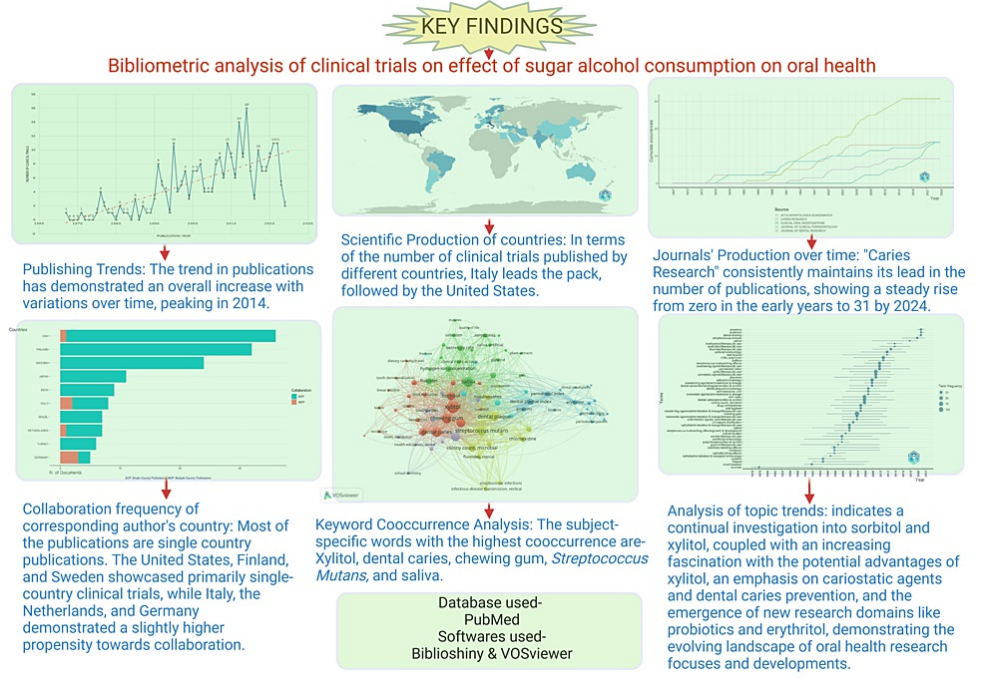
Key findings of the bibliometric analysis of clinical trials on the effect of sugar alcohol on oral health. This figure is generated using the premium version of the Biorender App [22] with the License Agreement Number KR26RJCLVH. Image credit: Namrata Dagli

This study represents a pioneering endeavor in conducting an extensive bibliometric analysis of clinical trials investigating the impact of sugar alcohol on oral health. No comparable analysis has been undertaken in this field, highlighting the originality and importance of this research initiative. This bibliometric analysis provides valuable insights into the distribution of clinical trial publications on sugar alcohol and oral health, highlighting the pivotal role of crucial authors, institutions, journals, and countries in shaping the research landscape. These findings include the quantitative analysis of the research landscape and the qualitative thematic analysis for a holistic understanding of the subject. We have summarized the key findings in Figure 13.

Although this bibliometric analysis offers valuable insights into the research landscape concerning the influence of sugar alcohol on oral health, it is crucial to acknowledge several limitations. One such limitation is inherent in the selection of databases, with this analysis relying solely on PubMed data. Since PubMed predominantly indexes biomedical literature, it may not fully capture research from other relevant disciplines, potentially skewing the representation of research trends and thematic clusters, mainly if any study is published in a non-PubMed indexed journal. Moreover, while bibliometric analysis sheds light on publication trends, collaboration networks, and geographical distribution of research output, it does not directly evaluate the quality or impact of individual studies. Merely assessing publication quantity might not accurately gauge the significance or rigor of research in this domain. Additionally, due to the extensive number of research papers, individual paper quality was not evaluated for bias, and the inclusion of only English articles may introduce language bias.

Future study recommendations

While this analysis suggests a breadth of research areas already addressed in the study of sugar alcohol and oral health, there are opportunities for future investigations to enrich our understanding of this complex relationship further. One recommendation is to conduct longitudinal studies spanning various age groups to assess the long-term effects of sugar alcohol consumption on oral health outcomes, including dental caries and periodontal conditions. Additionally, integrating qualitative research methods, such as interviews or focus groups, can provide valuable insights into individuals' perceptions and behaviors of sugar alcohol consumption and oral hygiene practices. Furthermore, exploring novel intervention strategies, such as personalized oral health promotion programs tailored to individuals' dietary habits and sugar and alcohol intake, could offer promising avenues for improving oral health outcomes. Lastly, interdisciplinary research approaches that combine microbiological, behavioral, and clinical perspectives may provide a more holistic understanding of how sugar alcohols impact oral microbiota and overall dental health. By addressing these recommendations, future studies can advance our knowledge and inform evidence-based practices for promoting oral health in diverse populations.

## Conclusions

The analysis of clinical trial publications on sugar alcohol and oral health unveils a dynamic and multifaceted research landscape shaped by various factors. The publication trend has shown overall growth with fluctuations over time, reaching its highest point in 2014. Key authors, institutions, and journals are pivotal in driving this landscape, with notable contributors like Makinen KK and the University of Turku leading the way. Collaborative networks among authors and institutions underscore the global nature of research efforts, with diverse trajectories observed across different countries. Noteworthy contributions from countries like Italy, the United States, Finland, Germany, and Canada showcase varying levels of engagement in clinical trial publications, with trends indicating steady growth or sporadic increases over time. Caries Research emerged as the most contributing journal. The thematic analysis reveals a comprehensive exploration of the subject utilizing various methodologies to assess efficacy, safety, and microbial dynamics. The study of topic trends reveals a continuous investigation into sorbitol and xylitol, with a rising interest in the latter's potential benefits, alongside a significant focus on cariostatic agents and dental caries prevention, and the emergence of new research areas such as probiotics and erythritol, highlighting the evolving landscape of oral health research priorities and advancements. This study provides valuable insights into the quantitative and qualitative aspects of research in this domain, guiding future priorities and interventions to promote oral health and well-being globally.
